# Ventilation with hyperoxia promotes cochlear bleeding in rabbits with congenital diaphragmatic hernia

**DOI:** 10.1016/j.clinsp.2024.100525

**Published:** 2024-11-06

**Authors:** Eduardo Tanaka Massuda, Solange Natalia Seibert, Ana Maria Bicudo Diniz, Luiza Almeida Lima, Maria Rossato, Vanessa Maciel Bráulio da Fonseca, Marcos de Carvalho Borges, Jason Xia, Amaury Lelis Dal Fabbro, Lourenço Sbragia

**Affiliations:** a Division of Otolaryngology, Department of Ophthalmology, Otolaryngology and Head and Neck, Faculdade de Medicina de Ribeirão Preto, Universidade de São Paulo, Ribeirão Preto, SP, Brazil; b Division of Pediatric Surgery and Anatomy, Department of Surgery and Anatomy, Faculdade de Medicina de Ribeirão Preto, Universidade de São Paulo, Ribeirão Preto, SP, Brazil; c Division of Pneumology, Department of Internal Medicine, Faculdade de Medicina de Ribeirão Preto, Universidade de São Paulo, Ribeirão Preto, SP, Brazil; d Division of Pediatric Surgery, Nationwide Children's Hospital, Columbus, OH, USA; e Department of Social Medicine, Faculdade de Medicina de Ribeirão Preto, Universidade de São Paulo, Ribeirão Preto, SP, Brazil

**Keywords:** Congenital Diaphragmatic Hernia, Cochlea, Sensorineural Hearing Loss, Damage

## Abstract

**Objective:**

The authors hypothesized that ventilation and hyperoxia may harm the cochlea vasculature in an experimental model of Congenital Diaphragmatic Hernia (CDH) performed in rabbits.

**Methods:**

New Zealand rabbits underwent CDH creation at 25 days of gestation (term = 30 days). CDH was created in fetuses (n = 15) and compared with Controls (n = 15). Six groups were studied: Control, Control Ventilated 21% FiO_2_ (Control 21%), Control Ventilated 100% FiO_2_ (Control 100%), CDH, CDH Ventilated 21% FiO_2_ (CDH 21%) and CDH Ventilated 100% FiO_2_ (CDH 100%). Dynamic Compliance (CRS), dynamic Elastance (ERS), and dynamic Resistance (RRS) were measured. The cochleae were then removed, and the apical, middle, and basal slopes of the cochleae were evaluated. Samples were graded using a scoring system for the severity of bleeding: 0 (absent), 1 (mild), 2 (moderate), and 3 (severe). Statistical analysis was performed by contingence and ANOVA.

**Results:**

There was no difference in the severity of cochlear bleeding between Controls and CDH without ventilation. Control 21% and 100% had average scores of 2 and 1.2 respectively; CDH 21% and 100% had average scores of 0.4 and 3.8 respectively; RR [CDH 100% / Control 100%] (95% CI) = 3.16 (p < 0.005).

**Conclusions:**

The severity of bleeding was 3.16 times worse with 100% oxygenation in CDH. This information may be helpful for future therapeutic strategies for decreasing SNHL in CDH patients.

## Highlights

• Congenital Diaphragmatic Hernia (CDH) is a congenital defect affecting 1 in every 5000 live births.
• Sensorineural Hearing Loss (SNHL) is an important prevalent complication in survivors.
• Mechanical ventilation and many other CDH therapies have been linked to SNHL.
• Cochlear bleeding may be responsible and explain part of SNHL in CDH ventilated patients.

## Introduction

Congenital Diaphragmatic Hernia (CDH) is a prevalent congenital anomaly that affects 1 in every 2500‒3000 live births. Advances in prenatal diagnosis and management have improved survival rates of infants with CDH over the years, but the survivors often suffer from long-term morbidities.^1^^,^^2^ Consequences of CDH can include pulmonary hypertension, neurodevelopmental sequelae, gastroenterological dysfunctions, and psychomotor disability.^3^ Some of the morbidities are not directly related to CDH but are due to the therapies instituted in perinatal care.

Sensorineural Hearing Loss (SNHL) is defined as hearing loss of the inner ear or dysfunction of the nerve pathways from the inner ear to the brain. Due to the improvement in neonatal care and the widespread implementation of SNHL immunization programs, it has been observed more and more frequently. Non-genetic risk factors for hearing loss during the neonatal period include treatment in a neonatal intensive care unit, craniofacial anomalies, and meningitis.^4^, ^5^, ^6^

SNHL is an important prevalent complication in CDH survivors, as it can lead to neurodevelopmental delay if not detected in a timely fashion.^7^ Significant SNHL (PTA ≥40 dB) was associated with markers of more severe CDH with a more complicated neonatal hospital course.^8^ The large discrepancy in these results can be justified by the development of new management strategies and by the health protocols where the study was developed.

The mechanism behind why the CDH population seems to be more affected by SNHL remains elusive. While some focus on the possible impact of postnatal intervention, other studies propose that severe hypoxia may lead to cochlear damage.^9^ Others have suggested that, since the development of the diaphragm and the cochlea occur at the same gestational age, the dysfunction of both organs may be related.^10^ The aim of this study is to evaluate the impact of ventilation on the cochlea in an experimental model of CDH.

## Methods & methods

The ethics committee of studies in animal experimentation approved the experiment at the Ribeirao Preto Medical School, University of Sao Paulo (CEUA – FMRP-USP, protocol # 1002/2021R2).

### *Experimental groups*

Pregnant New Zealand Rabbits (term = 30-days) were divided into 6 experimental groups with (n = 5 each): 1) Control (C): rabbit pups born at 30-days of gestation without experimental intervention; 2) Control 21% (Control Ventilated 21% FiO2); 3) Control 100% (Control Ventilated 100% FiO2); 4) Congenital Diaphragmatic Hernia (CDH): fetuses of pregnant rabbits with left CDH creation on the 25^th^ day of gestation and harvested on the 30^th^ day of gestation; 5) CDH 21% (CDH Ventilated 21% O2) and 6) CDH 100% (CDH Ventilated 100% O2).

### *CDH creation*

The procedure was performed according to Fauza D. et al. on gestational day 25.^11^ Under general anesthesia and prophylactic antibiotic therapy, the uterus was exposed through a midline laparotomy. After identification of the fetal position, a purse-string suture with 5‒0 Prolene® was performed at the level of the fetal thorax, which was seen by direct visualization. A hysterotomy was performed with careful avoidance of the lateral uterine vessels and the left forelimb was exposed outside the uterine cavity. A 3 mm incision was created with microsurgical scissors to enter the thoracic cavity. The diaphragmatic muscle was gently grasped with micro forceps and an incision was made to create a diaphragmatic hernia. After the incision, the thorax was closed with 6‒0 Prolene®. At the end of the experiment, 2 mL of warm saline was administered into the amniotic cavity prior to the closure of the uterine wall to replace amniotic fluid losses. The uterine incisions were closed in one layer with 2‒0 Vicryl® in a continuous fashion, and the abdominal wall was closed with 4‒0 Nylon® in a continuous intradermal fashion. At the end of the surgery, 25 mg/kg of Cefazolin Sodium and 2.5 mg/kg of Medroxyprogesterone Acetate (Depo Provera®) were administered intramuscularly to prevent fetal abortion and surgical infection.

After surgery, the rabbit was maintained at a consistent SpO2 and temperature for 1‒2 hours to assist in recovery from the surgical procedure. The fetuses were harvested on gestational day 30 with the same anesthetic protocol. The experimental and control fetuses were removed, cleaned, and weighed. After specimen collection, the anesthetized rabbit was sacrificed with a lethal dose of sodium thiopental.

### *Mechanical ventilation and measurement of in vivo parameters of pulmonary function*

On gestational day 30, the rabbits were anesthetized by intramuscular injection of S (+) ketamine hydrochloride (ketamine®) at a dose of 25 to 50 mg/kg (1 vial = 50 mg/mL) and Xylazine (Rompun® ‒ Bayer) at a dose of 5 to 10 mg/kg (1 vial = 200 mg/mL) in the right posterior thigh. Median laparotomy was performed to expose the uterus and experimental animals were removed. The neonates were weighed and a dose of 25 to 50 mg/kg of ketamine was given intraperitoneal. A tracheostomy was performed for endotracheal intubation. After an injection of Lidocaine hydrochloride 0.5% (5 mg/kg, SC) at the midline neck. They were positioned in dorsal decubitus on a heated table, where they underwent an anterior cervical incision and isolation of the trachea. Tracheal intubation was performed using an 18G Teflon (BD) intravascular catheter 1.2×40 mm that was connected to a small FlexiVent animal ventilator (Scireq, Montreal, QC, Canada). Ventilator The settings of the ventilator were: the respiratory rate at 150 breaths/minute, PEEP (Positive End-Expiratory Pressure) at 4 cm H2O, inspiratory time at 0.1s, and expiratory time at 0.3s.^12^ Animals were ventilated with 21% or 100% fraction of inspired oxygen as designated by the group. Lung measurements were recorded every 4 minutes for up to 24 minutes. The temperature was controlled via a thermal mattress and animals were wrapped in plastic for additional thermal maintenance, with measurements taken every 10 minutes. All the neonates survived until the end of the experiment.

### *Morphometrics analysis*

To evaluate the effect of CDH on lung growth, Body Weight (BW) and Total Lung Weight (TLW) were measured, and the TLW/BW ratio was calculated.

### *Cochlear removal*

Rabbits were immediately sacrificed after removal of the uterus for the non-ventilated group and after ventilation for the ventilated group. Next, the rabbits were decapitated, and a medial sagittal craniotomy was performed, separating the two hemicraniums. Under 4× microscopic dissection (DFV – Vasconcelos, Valença, RJ, Brazil). Subsequently, the temporal bones were isolated by removing the remaining bone portions of the cranial structure. The mastoid bulla was opened, thus exposing the cochlea.

After removal, the cochleae were incubated in a 3% glutaraldehyde fixation solution at 4 °Celsius for 24 hours. A 3% glutaraldehyde solution in 0.1M phosphate buffer, pH = 7.4, was injected into the cochlea through the round window and incubated for 4 hours at 4 °Celsius. The cochleae were then washed three times for 5 minutes each with the same buffer solution and fixed with 1% osmium tetroxide for 2 hours at 4 °Celsius. Subsequent dehydration occurred at room temperature using increasing concentrations of ethanol (50%, 70%, 90%, and 95% ‒ once, for 10 minutes at each concentration, and 100% ethanol three times for 15 minutes each). After dehydration, it was dried using the CO2 critical point method. After decalcification, H&E staining was used to verify the degree of cochlear bleeding. 5-micrometer cuts were performed to assess cochlear damage.

### *Cochlear bleeding assessment scale*

An assessment scale was created to determine the severity of bleeding in the basal ramp, mid ramp, and apical ramp: 0: Absent; 1: Mild (Bleeding affects < 20% of the area); 2: Moderate (Bleeding affects 20%‒50% of the area); and 3: Severe (Bleeding affects > 50% of the area). Three blinded experimental raters analyzed each section before cochlear bleeding standardization training. Scores were recorded separately by the three examiners (ETM, MR, LS) and assigned arbitrary values (au) according to signs of bleeding. Then, an average score was calculated for each histological section.

### *Statistical analysis*

Statistical comparisons were made using one-way analysis of variance (ANOVA) and the Bonferroni method was used as a post hoc test; p < 0.05 was considered statistically significant. The program used for statistical data analysis was GraphPad, version 8.0 (GraphPad Prism Software, San Diego, CA, USA).

## Results

A total of 10 pregnant rabbits were used. The average weight was 3.442 g (3310 – 3788 g). The total survival was 100% for Control, Control 21% and Control 100% (n = 15 each, total 15). The total survival was 65% for CDH, CDH 21% and CDH 100%, respectively 5 out 7; 5 out 9; and 5 out 8 (n = 15 each, total 24). All surgically created CDHs had hole sizes classified as C and D according to the CDH Study Group Consensus.

### *The morphometrics*

**Fig. 1:**
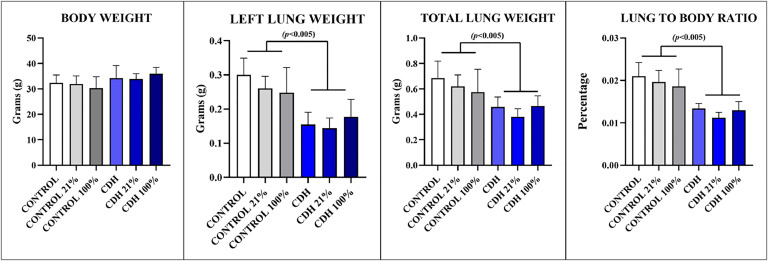
Results of morphometric measurements among the groups.

Body Weight (BW): Control – 32.35 g (±3.12), Control 21% ‒ 31.85 g (±3.31), Control 100% ‒ 30.35 g (±4.47), CDH – 34.25 g (±4.92), CDH 21% ‒ 33.87 g (±2.12), CDH 100% ‒ 35.96 g (±2.52). There was no statistical difference among groups (NS). Left Lung Weight (LLW): Control – 0.300 g (±0.049), Control 21% ‒ 0.261 g (±0.036), Control 100% ‒ 0.248 g (±0.073), CDH – 0.155 g (±0.036), CDH 21% ‒ 0.144 g (±0.030), CDH 100% ‒ 0.177 g (±0.051). Total Lung Weight (TLW): Control – 0.685 g (±0.134), Control 21% ‒ 0.621 g (±0.089), Control 100% ‒ 0.576 g (±0.179), CDH – 0.458 g (±0.078), CDH 21% ‒ 0.380 g (±0.064), CDH 100% ‒ 0.466 g (±0.080). Lung to Body Ratio (LBR): Control – 0.021 (±0.003), Control 21% ‒ 0.020 (±0.003), Control 100% ‒ 0.019 (±0.004), CDH – 0.013 (±0.001), CDH 21% ‒ 0.011 (±0.001), CDH 100% ‒ 0.013 (±0.002). With all ventilatory groups combined, there was a significantly lower LLW in CDH rabbits when compared to Controls (p < 0.005). When subdivided by the ventilatory group, 21% FiO2 had a higher left lung weight compared to 100% FiO2 in the control population, but the opposite was seen in the CDH population. The same differences between control animals and CDH animals, with all ventilatory modes combined, were noted for TLW (p < 0.005) and LBR (p < 0.005). The differences seen in LLW when subdivided by the ventilatory group were also the same for TLW and LBR (NS) (Fig. 1).

### *Mechanical ventilation and in vivo lung ventilatory parameters*

**Fig. 2:**
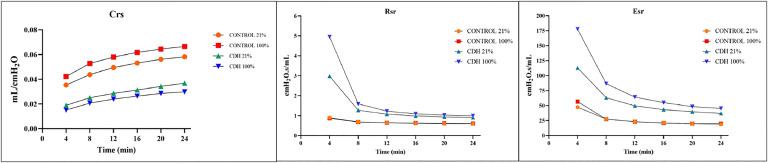
Results of Complacence, Resistance and Elastance among the groups. Crs, Dynamic compliance; Rrs, Dynamic resistance; Ers, Dynamic elastance. Control 21% and Control 100% (p < 0.05) in Ers. Crs and Rrs were different between Controls and CDHs (p < 0.005). There was no difference between CDH 21% and CDH 100% for Crs, Rrs, and Ers.

With all ventilatory groups combined, Crs was significantly higher among the Control animals compared to the CDH animals (p < 0.05). Rrs and Ers were both found to be significantly lower among Control animals compared to CDH animals (p < 0.005). Crs was also found to be significantly lower in the Control 21% group compared to the Control 100% group. No statistical difference was found among ventilatory groups for Rrs and Ers (Fig. 2).

### *Cochlea bleeding score*

**Fig. 3:**
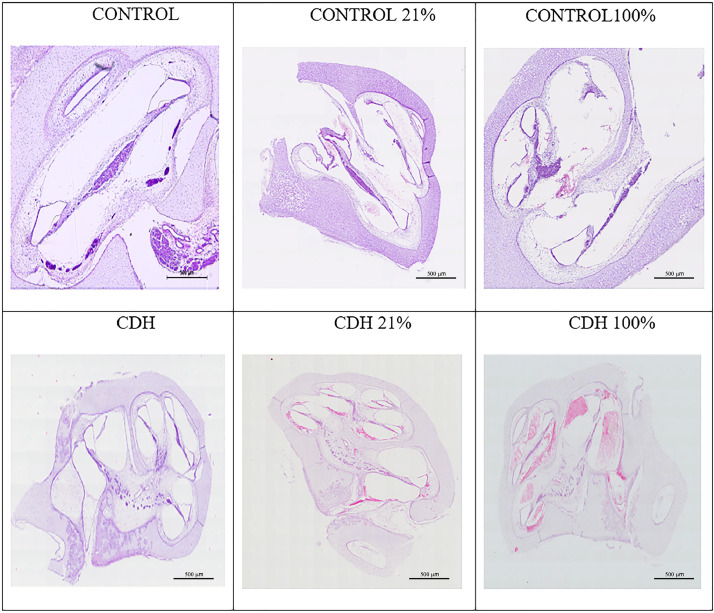
**Photomicrograph** of cochlear bleeding among the groups.

The cochleae from 5 animals for each group were studied with the average score (Table 1 and Fig. 3). Control and CDH groups without ventilation did not present any bleeding.

**Table 1:** Cochlea Bleeding Score of Control and CDH 21% and 100%.

	VENT 21%	VENT 100%	p
**Control**	2.0 (95% CI 1.44‒2.55)	1.2 (95% CI 0.47‒1.92)	0.0218^a^
**CDH**	0.4 (95% CI 0.10‒0.69)	3.8 (95% CI 2.73‒4.86)	0.0003^b^
**p**	0.0002^c^	0.0004^d^	

a Control 21% vs. Control 100%.

b CDH 21% vs. CDH 100%.

c Control 21% vs. CDH 21%.

d Control 100% vs. CDH 100%.

## Discussion

SNHL is an important complication in CDH survivors, as it can lead to neurodevelopmental delay if not detected in a timely fashion. Although the incidence of childhood SNHL has declined in most developed countries, the risk of bilateral SNHL for newborns in the neonatal intensive care unit is up to ten times greater in the absence of a family history or an identifiable syndrome. Most of the risks are associated with prematurity treatment, aminoglycosides, mechanical ventilation, and hyperbilirubinemia due to anemia.^13^ However, additional acquired causes include congenital infections (rubella and cytomegalovirus), ototoxicity, prematurity, asphyxia, and others.^4^

The authors previously evaluated the vascular ventilatory response in different stages of lung development E25, E27, E30 (term rabbit = 30‒31 days) and compared them to the neonates with Congenital Diaphragmatic Hernia (CDH). CRS, ERS and RRS were measured every 4 min/24 min. Median Wall Thickness (MWT) and airspace were measured. The ventilation response of CDH is like the pseudo glandular stage of lung development instead alveolar stage. MWT was decreased according to the gestational age, was increased in E27V and E30V (p < 0.05), and decreased in CDHV (p < 0.05). These findings add information about the physiology of pulmonary ventilation in CDH and show a decreased pattern of lung development with a ventilatory response that is hypoxemic.^14^

The prevalence of SNHL in the CDH population is still debated as it can vary from 2% to 60% of all CDH survivors who have late-onset progressive SNHL. The great discrepancy in these results can be explained by the development of new management strategies, by the health protocols for the identification of hearing loss, and by the patients’ follow-up time.^2^ This study focused on ventilatory management strategies as a possible contributor to SNHL in the CDH population, with multiple significant findings. In this experimental model, the lung compliance for the Control 21% group was significantly lower than that of the Control 100% neonates. There were no differences between the parameters for compliance, resistance, and elastance for concentrations of 21% and 100% in CDH. However, there were differences for all parameters between neonates with CDH and controls regardless of FiO2.

The authors also found cochlear bleeding scores to be higher in the Control 21% neonates compared to the Control 100% neonates, suggesting that hyperoxia attenuates the severity of cochlear bleeding in a normal lung. This finding is in line with previous literature, and the vulnerability of the cochlea to reduced oxygen supply has been most demonstrated in acute electrophysiological experiments.^15^ Controlled hypoxia in the guinea pig model has been shown to damage hair cells, resulting in complex biochemical changes within the cochlea. Additionally, carbon monoxide (CO) has been shown to result in pH shifts within the cochlear fluids and increase the risk of bleeding. Even short-term asphyxia may cause a drop in the amount of oxygen in the perilymph and endolymph compromising the cochlea, which is highly oxygenated under normal conditions.^16^ The authors did not investigate the organ Corti, however these findings indirectly show cochlear bleeding as part of hypoxic damage in CDH and mechanical ventilation in control groups. The present results are inconsistent with the findings in those authors, but no less valid. Although the organ of Corti is more resistant to hypoxia because it has more energy reserves, the stria vascularis, an area of high aerobic metabolism, may be even more vulnerable to bleeding.^17^

An experimental study of cochleae of newborn rats exposed to increased CO during pregnancy and after birth showed greater cochlear damage. The hypoxic process generates oxidative stress in the cochlear vessels and promotes damage in the spiral ganglia. Such findings suggest that poor management of ventilation with increased CO supply can cause damage to the inner ear.^18^

Additionally, the cochlea is sensitive to chemical toxins, especially in developing mammals. Coenzyme Q10 (CoQ10) is a potent antioxidant and is effective in recovering from acute deafness. In a study of CoQ10-treated guinea pigs, acute SNHL loss was artificially induced by hypoxic conditions and resulted in a gradual disappearance of the Auditory Brainstem Response (ABR). Treated cochleae recovered from damage to the auditory hairs and were protected from the characteristic metabolic impairment of hair cells due to hypoxia.^19^ Another study in newborn and young rats showed that the cochlea of younger rats has immature complements of detoxification enzymes, which explains the differences in susceptibility to ototoxic agents and therefore greater susceptibility to the use of antibiotics.^20^

Interestingly, neonates with CDH had the opposite, with higher bleeding severity scores in the 100% FiO2 group compared to the 21% FiO2 group. Additionally, the CDH 21% group had less severe bleeding compared to the Control 21% group. These findings are suggestive of lower oxygen supply being a protective factor in CDH, although the underlying mechanism for this is unclear. This information may be helpful for future therapeutical strategies applying drugs with the protection of oxidation–reduction reaction for decreasing the possible SNHL.

In addition to mechanical ventilation, many other CDH therapies, known to improve cardiac function and nutritional status, have been linked to SNHL.^21^ The survivors of CDH are highly exposed to neonatal risk factors for SNHL, such as hypocarbia before and after ECMO,^22^ long use of aminoglycoside antibiotic therapy and diuretics (furosemide),^23^ neuromuscular blocking agents, inhaled nitric oxide, and High-Frequency Oscillation (HFO).^24^ In a study with 112 patients with CDH, 2.7% had SNHL and 34% had Conductive Hearing Loss (CHL). SNHL was significantly associated with the requirement for ECMO, duration of mechanical ventilation, duration of loop diuretic, aminoglycoside therapy, the requirement for tracheostomy, and prolonged course of hospitalization. In a retrospective study of 55 patients with CDH evaluated by means of the Pure-Tone Audiogram (PTA) and classified from normal to profound SNHL, it was demonstrated the same variables already described for risk of SNHL, in addition to non-primary repair of CDH. Moderate levels of PTA (≥ 40 dB) were associated with significant SNHL for more severe CDH and more complicated neonatal hospitalizations.^8^ Overall, the prevalence of SNHL in infants who underwent ECMO therapy is 8.1%‒26%.^9^, ^25^ However, some studies show that those with a primary diagnosis of CDH were 2.6 times more likely to develop SNHL after ECMO therapy than the general population, which could indicate that the CDH population is more susceptible to SNHL.^26^ These studies highlight the need for audiological monitoring of speech and language development for survivors with severe CDH.^27^

The present study is the first to study the effect of varying ventilatory strategies in a large animal model of surgically induced CDH. Large animal models of CDH have multiple advantages; they have increased feasibility for treatments and intervention, such as tracheal occlusion, and they are easier to handle.^28^ By using this surgical model, the authors are also able to mimic lung underdevelopment by the dual hit hypothesis, like in human neonates. The first insult occurs by failure to form the diaphragm muscle, and the second hit is due to the growth of abdominal organs (intestine, stomach, and liver) into the thorax, interrupting lung growth.^29^

The limitations of this study include the novelty of the animal model and the short duration of ventilation. Although the authors used a well-established rabbit model of CDH, most experimental studies on the cochlea were done in guinea pigs. Additionally, arterial blood gas assessment was not verified, and the status of cochlear damage was restricted to a short period of time on ventilation while most infants with CDH remain on ventilation for a long time.

In conclusion, this study is the first to demonstrate the effect of ventilation on cochlear bleeding as a potential mechanism for SNHL in CDH patients. These results show that in a neonatal rabbit CDH model, 100% FiO2 significantly increases the severity of cochlear bleeding compared to 21% FiO2. Additionally, lung resistance and elastance were significantly elevated in CDH animals compared to controls. These results suggest that SNHL in CDH may be directly related to ventilatory strategies for neonates in the NICU and is related to the underlying pulmonary development. Further studies will need to elucidate these management strategies as potential causative factors for SNHL in CDH patients, and these results will be helpful in the development of future therapeutic strategies to protect against SNHL in this vulnerable population.

## Authors’ contributions

Eduardo Tanaka Massuda: Investigation, data curation (histological cochlea classification), writing-review & editing.

Solange Natalia Seibert: Investigation, data curation (surgery).

Ana Maria Bicudo Diniz: Investigation, data curation (surgery), writing-review & editing.

Luiza Almeida Lima: Investigation, data curation (surgery).

Maria Rossato: Investigation, data curation (histological cochlea preparation).

Vanessa Maciel Bráulio da Fonseca: Investigation, data curation (ventilation).

Marcos de Carvalho Borge: Investigation, data curation (ventilation), writing-review & editing.

Jason Xia: Methodology writing-review & english editing.

Amaury Lelis Dal Fabbro: Investigation, data curation, statistical analysis.

Lourenço Sbragia: Conceptualization, investigation, project administration (surgery), writing-review & editing.

## Declaration of competing interest

The authors declare no conflicts of interest.
